# Risk of low bone mineral density in patients with haemophilia: a systematic review and meta-analysis

**DOI:** 10.1186/s13018-023-04499-6

**Published:** 2024-01-11

**Authors:** Haojing Zhou, Lei Chen, Hai Su, Guoqian Chen, Peijian Tong

**Affiliations:** 1 Zhejiang Chinese Medical University, Hangzhou, Zhejiang Province China; 2 The First Affiliated Hospital of Zhejiang Chinese Medical University (Zhejiang Provincial Hospital of Chinese Medicine), Hangzhou, Zhejiang Province China

**Keywords:** Bone density, Haemophilia, Meta-analysis, Osteopenia, Osteoporosis

## Abstract

**Introduction:**

Patients with haemophilia (PWH) may have lower bone mineral density (BMD). The risk of low BMD in PWH has not been comprehensively analysed. This study aimed to examine the risk of low BMD and changes in BMD in PWH.

**Methods:**

A comprehensive systematic search was performed in 4 databases: PubMed, Embase, Web of Science, and Cochrane Library. The last search was carried out on 11 December 2022. Review Manager 5.4 and Stata 16 were used for meta-analysis. Odds ratios were calculated by the incidence of low BMD between the haemophilia and control groups in each study. A meta-analysis of the odds ratios for each study was performed to estimate pooled odds ratios. Fixed effects models or random effects models were used to assess outcomes. Heterogeneity was evaluated using Higgins’ I^2^. Subgroup analysis and sensitivity analysis were performed to interpret the potential source of heterogeneity. A funnel plot, Egger’s regression test, and the trim-and-fill method were used to assess publication bias.

**Results:**

19 of 793 studies, published between 2004 and 2022, that were identified by search strategy were included in this meta-analysis. The risk for low BMD was approximately four times higher compared to controls. PWH have significantly lower lumbar spine, femoral neck, and total hip BMD. Subgroup analysis showed that the risk of low BMD did not increase significantly in developed countries. Very low heterogeneity was observed in the meta-analysis of the risk of low BMD. The result from Egger’s regression test suggested that there may be publication bias. However, the meta-analysis results did not alter after the trim-and-fill correction and the findings were robust.

**Conclusion:**

Haemophilia was associated with an increased risk of low BMD. However, the risk of low BMD did not increase significantly in developed countries. And BMD was reduced in PWH, regardless of age, region, or economic ability. For PWH, our concerns should extend beyond bleeding and osteoarthritis to encompass BMD starting at a young age.

**Supplementary Information:**

The online version contains supplementary material available at 10.1186/s13018-023-04499-6.

## Introduction

Haemophilia A and B are X-linked recessive disorders caused by lack or deficiency of clotting factor VIII (FVIII) or IX (FIX) and primarily affect male patients. Bleeding, particularly in the knee, ankle, and elbow joints, is the hallmark clinical manifestation of haemophilia and can result in arthropathy [1]. Haemophilia severity is classified by the amount of residual FVIII or FIX activity: severe (< 1 IU/dl), moderate (1–5 IU/dl), and mild (6–40 IU/dl) [2]. The prevalence of haemophilia A and B is 17.1 and 3.8 cases per 100,000 males, respectively. The estimated number of global patients with haemophilia (PWH) is 1,125,000, of which 418,000 are severe haemophilia [3].

Low bone mineral density (BMD) is a condition in which increased bone resorption, decreased bone formation, or a combination of both, leading to reduced bone mass [4]. It is reported that the prevalence of osteoporosis was 11.7% among males worldwide [5]. Historically, the prevention and treatment of osteoporosis in men have frequently been overlooked. Although osteoporosis is more prevalent among women, men experience greater disability and mortality than women before the age of 65 [4]. Urgent attention is needed for addressing the management of low BMD in men.

BMD is assessed through dual X-ray absorptiometry (DXA), an excellent tool with low radiation exposure [6]. The World Health Organization (WHO) international reference standard for osteoporosis diagnosis is a T-score of − 2.5 or less in men over the age of 50 and postmenopausal women. And according to the International Society for Clinical Densitometry (ISCD), the Z-score is recommended for patients under 50 years of age. A Z-score of − 2.0 or lower is defined as “below the expected range for age” and a Z-score above − 2.0 is “within the expected range for age”. The WHO diagnostic criteria applied to women in the menopausal transition. Osteoporosis should not be diagnosed in men under age 50 based on BMD alone [7]. Consequently, we categorized osteoporosis and “below the expected range for age” as low BMD in this study.

In developed countries, the primary treatment approach for haemophilia involves administering regular injections of clotting factors to prevent bleeding episodes. However, this practice is not prevalent in many developing countries due to inadequate healthcare infrastructure, budgetary limitations, and other factors [8]. Bleeding may lead to lower BMD [9]. As far back as 1994, Gallacher [10] identified lower BMD in PWH compared to the general population. Currently, two studies [11, 12] have conducted meta-analysis on changes in BMD, which showed that PWH presented a significant reduction in both lumbar spine and hip BMD compared to the general population. But these studies did not evaluate whether the extent of BMD decline fulfilled the criteria for low BMD. In other words, the comprehensive analysis of the risk of low BMD in PWH has not been conducted.

Therefore, we conducted a comprehensive systematic review and meta-analysis to evaluate the risk of low BMD in males and children with haemophilia A and B. This meta-analysis was based on odds ratios, allowing for both quantitative and qualitative comparisons against the general population. Furthermore, we explored the potential factors contributing to reduced BMD in PWH.

## Materials and methods

This systematic review and meta-analysis followed the Meta-analysis Of Observational Studies in Epidemiology (MOOSE) guidelines [13], and reference to the Preferred Reporting Items for Systematic Reviews and Meta-analysis (PRISMA) statement [14]. The review protocol was registered in the PROSPERO database (registration number: CRD42017060022).

### Data sources and searches

A comprehensive systematic search of the following 4 databases was performed: Pubmed, Embase, Web of Science, and Cochrane Library. The last search was carried out on 11 December 2022. References to the included studies were also browsed for potentially relevant publications. The search strategy is detailed in Additional file 1: Table S1.

### Inclusion and exclusion criteria

Inclusion criteria for our meta-analysis were as follows: (1) Observational studies with a control or comparison group of age, body mass index (BMI), and sex-matched population without haemophilia; (2) Low BMD was defined as BMD T-score of − 2.5 or less referring to WHO or BMD Z-score of − 2.0 or less referring to ISCD; (3) Articles reported numerical data of the prevalence of low BMD or changes in BMD in haemophilia versus non-haemophilia groups; (4)Adult men and children; (5)Haemophilia A or haemophilia B; and (6) Receiving replacement therapy with clotting factor either on-demand or as prophylaxis.

Exclusion criteria for our meta-analysis were as follows: (1) Articles written in non-English; (2) Articles were animal or cell line studies; (3) The type of articles were conference reports, case reports, or reviews; (4) Other bleeding disorders; and (5) Acquired haemophilia.

### Study selection and data abstraction

Two authors independently reviewed titles and abstracts to further identify potentially eligible studies. Disagreements were discussed with a third author. Information from each study was extracted independently by two authors using a standardized data extraction form. The following data were extracted: first author, publication year, region, mean age, BMI, the prevalence of low BMD, and different sites of BMD in PWH and control groups. The highest data are selected when a study reported the prevalence of low BMD in multiple sites. If necessary, the corresponding authors were contacted for additional information.

### Quality assessment

The Newcastle–Ottawa Scale (NOS) for assessing the quality of non-randomized controlled trials in the meta-analysis was used [15]. It is formulated by assigning a maximum of nine stars to studies of the highest quality according to three parameters: selection, comparability, and exposure. In NOS, the score ranged from 0 to 9, where a score of 9 indicates the strongest regarding methodology. Low-, moderate- and high-quality studies were scored 0–3, 4–6, and 7–9, respectively.

### Statistical analysis

All statistical analysis in this meta-analysis was conducted using Review Manager 5.4 and Stata 16.0 statistical software. When the exact number of low BMD events was available, meta-analysis was performed to assess odds ratios (OR) for each studied group to estimate pooled OR with their 95% confidence intervals (CI). Standardized mean differences (SMD) with 95% CI for changes in BMD in haemophilia groups versus controls were calculated. Only the data presented as mean ± standard deviation (SD) will be analysed.

Heterogeneity is assessed by Higgins *I*^2^ statistic and 0–25% suggests very low heterogeneity, 25–50% low heterogeneity, 50–75% moderate heterogeneity, and more than 75% high heterogeneity [16]. The fixed effects models would be enabled if *I*^2^ < 50%; otherwise, a random effects model was applied. And subgroup analysis or sensitivity analysis was performed to interpret the potential source of heterogeneity. Sensitivity analysis was used to test the robustness of significant results.

To provide a visual inspection of publication bias, the funnel plot was generated, and to examine publication bias quantitatively, Egger’s regression test was employed. If there is a publication bias, we will use the trim-and-fill method to correct it.

The authors performed the statistical analyses.

## Results

### Study selection

**Fig. 1:**
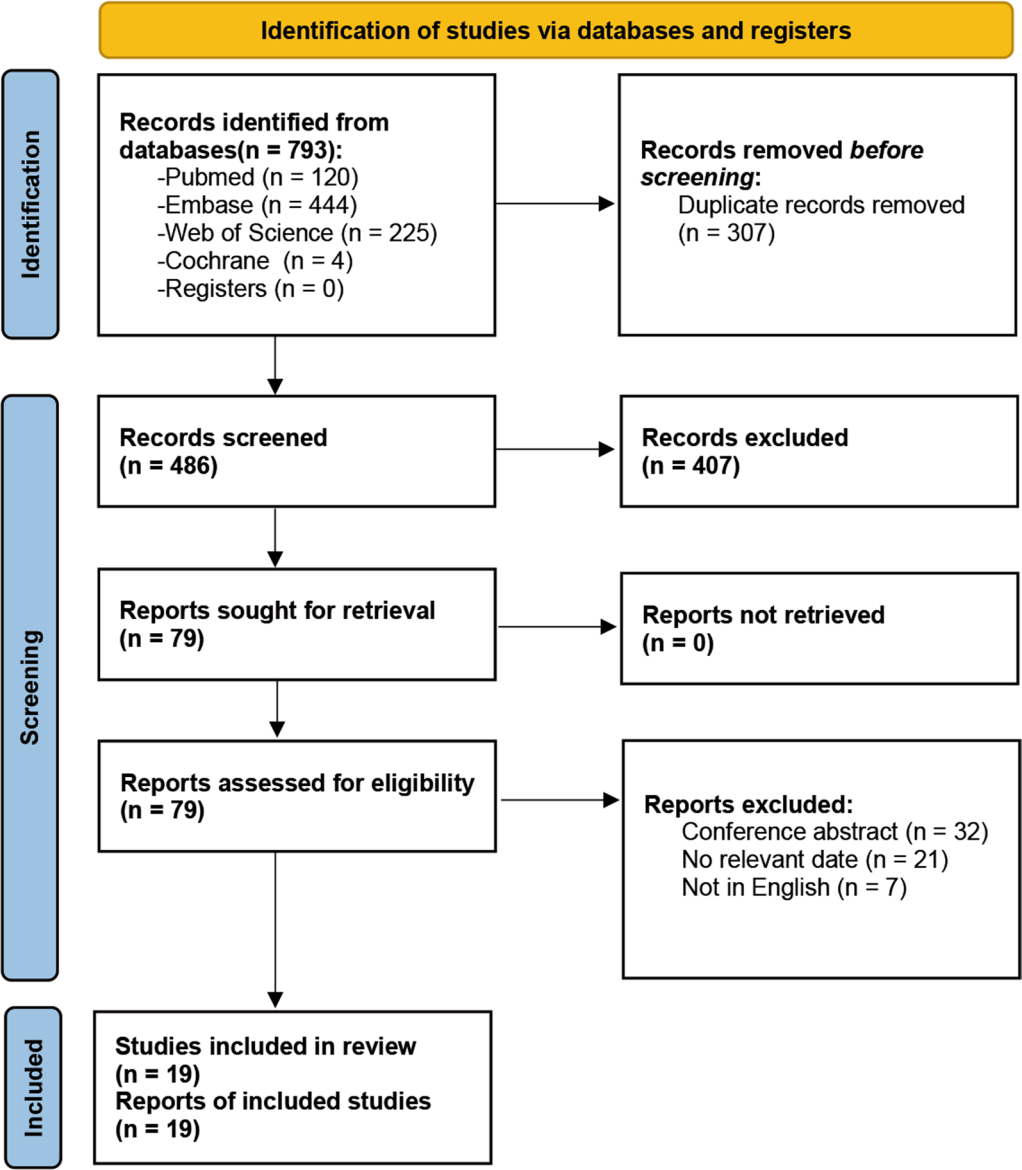
Flow diagram of study selection

The study selection process is presented in Fig. 1. A total of 793 relevant references were identified in our initial search, and after removing any duplicates, 486 records were identified as potential references. We screened the titles and abstracts of all the references and identified 79 studies for full-text review. After a full review of these 79 studies, 19 studies [17–35] met the inclusion criteria for systematic review and meta-analysis. The studies were published between 2004 and 2022.

### Study characteristics

Tables 1 and 2 show the basic characteristics and main outcomes of the included studies. All eligible studies were published in English. Of these included studies, 13 reported prevalence of low BMD as an outcome [21–23, 26–35], and 16 reported changes in BMD as an outcome [17–22, 24–28, 30, 31, 33–35]. 16 studies [17–22, 24–28, 30, 31, 33–35] examined BMD of lumbar spine (LS), 6 [20, 22, 24, 26, 27, 31] examined BMD of femoral neck (FN), and 6 [19, 26, 27, 31, 33, 35] examined BMD of total hip (TH). 12 [17–22, 24–28, 34], 5 [20, 22, 24, 26, 27], and 3 [19, 26, 27] studies reported the data of LS, FN, and TH BMD presented as standard deviation, respectively. Of the included studies, 9 [19, 20, 22, 24, 25, 30, 31, 33, 35] recruited patients from Asia, 3 [23, 26, 27] from Europe, 4 [18, 28, 32, 34] from Africa, 1 [17] from Australia, 1 [21] from North America, and 1 [29] from South America. Among the included studies, in 10 studies [17, 18, 21, 23, 25, 28, 30, 32, 34, 35], mean age was less than 18 years and in 8 [19, 20, 22, 24, 26, 27, 31, 33] studies more than 18 years, and one study [29] did not report mean age. 4 [17, 23, 26, 27] recruited patients from developed countries and 15 [18–22, 24, 25, 28–35] from developing countries.

**Table 1:** Main characteristics of the studies included in the systematic review

Study	Year	Country	NO (H)	NO (C)	H type (A/B)	Mean age (H) (years)	Mean age (C) (years)	BMI (H)(kg/m^2^)	BMI (C)(kg/m^2^)	Infected (HBV/HCV/HIV)
Barnes, C	2004	Australia	19	215	19/0	12.2 ± 3.5	12.8 ± 2.1	N/A	N/A	NA/8/NA
Abdelrazik, N	2007	Egypt	30	30	21/9	4.97 ± 3.64	5.09 ± 3.64	16.76 ± 2.08	17.33 ± 2.19	0/0/0
Nair, A. P	2007	India	50	50	42/8	29.53 ± 9.27	29.20 ± 3.79	19.16 ± 3.8	23.62 ± 2.79	2/18/1
Mansouritorghabeh, H	2008	Iran	18	18	18/0	29.05 ± 8.62	29.05 ± 8.61	22.43 ± 3.30	23.64 ± 3.65	1/11/NA
Tlacuilo-Parra, A	2008	Mexico	62	62	62/0	9.02 ± 3.70	9.30 ± 3.70	18.40 ± 4.50	20.20 ± 5.70	N/A
Mansouritorghabeh, H	2009	Iran	14	14	0/14	30.57 ± 12.18	30.50 ± 12.14	25.37 ± 5.23	25.15 ± 4.89	NA/11/NA
Christoforidis, A	2010	Greece	26	13	26/0	12.08 ± 4.44	11.45 ± 5.26	N/A	N/A	NA/0/0
Rezaeifarid, M	2011	Iran	55	87	N/A	23.5 (Min:16, Max:35)	25.1 (Min:15, Max:35)	N/A	N/A	N/A
Alioglu, B	2012	Turkey	44	40	44/0	160months (Min:36, Max:216)	146months (Min:42, Max:240)	N/A	N/A	N/A
Anagnostis, P	2012	Greece	104	50	90/14	45.87 ± 15.15	44.90 ± 12.80	27.05 ± 4.51	28.49 ± 4.39	4/42/7
Wells, A. J	2015	UK	37	37	37/0	31 (IQR:13–49)	35 (IQR:23–47)	25.4 (IQR:19.7–31.1)	26 (IQR:22.6–30.0)	NA/23/12
Eldash, H. H	2017	Egypt	37	37	N/A	8.3 ± 3.5	7.3 ± 2.2	N/A	N/A	0/0/0
Sossa Melo, C. L	2018	Colombia	90	90	79/11	N/A	N/A	N/A	N/A	N/A
Ashritha, A	2019	India	38	38	35/3	8.7 ± 3.6	8.7 ± 3.5	13.8 (IQR:12.9–15.2)	14.5 (IQR:13.6–15.5)	N/A
Ekinci, O	2019	Turkey	41	40	41/0	26 (Min:18, Max:56)	30 (Min:18, Max:54)	22.7 (Min:15, Max:30)	24.2 (Min:17, Max:32)	3/0/0
El-Mikkawy, D. M. E	2019	Egypt	40	10	40/0	10.98 ± 3.85	10.80 ± 3.49	18.64 ± 3.53	Matched	N/A
Ehsanbakhsh, A	2020	Iran	57	60	57/0	32.9 ± 77.32	34.8 ± 87.40	21.3 ± 49.89	22.4 ± 68.78	N/A
Mohamed, H. R	2020	Egypt	39	20	35/4	9.23 ± 4.36	9.40 ± 4.02	11.79 ± 3.90	12.30 ± 4.13	0/5/0
Patel, G. R	2022	India	39	40	35/4	9.0 (Min:1, Max:17)	9.5 (Min:1, Max:17)	13.8 (Min:10.6, Max:27.7)	14.5 (Min:11.4, Max:23.2)	0/0/0

*H* Haemophilia, *C* Controls, *BMI* Body mass index, *HBV* Hepatitis B virus, *HCV* Hepatitis C virus, *HIV* Human immunodeficiency virus, *N/A* Non-available

**Table 2:** Main outcomes of the studies included in the systematic review

Study	Low BMD (H)	Low BMD rate (H)	Low BMD (C)	Low BMD rate (C)	LS BMD (H) (kg/m^2^)	LS BMD (C) (kg/m^2^)	FN BMD (H) (kg/m^2^)	FN BMD (C) (kg/m^2^)	TH BMD (H) (kg/m^2^)	TH BMD (C) (kg/m^2^)
Barnes, C	N/A	N/A	N/A	N/A	0.72 ± 0.19	0.79 ± 0.19	N/A	N/A	N/A	N/A
Abdelrazik, N	N/A	N/A	N/A	N/A	0.48 ± 0.13	0.55 ± 0.14	N/A	N/A	N/A	N/A
Nair, A. P	N/A	N/A	N/A	N/A	0.82 ± 0.14	0.93 ± 0.11	N/A	N/A	0.73 ± 0.15	0.94 ± 0.11
Mansouritorghabeh, H	N/A	N/A	N/A	N/A	1.13 ± 0.11	1.29 ± 0.23	0.802 ± 0.23	1.45 ± 0.50	N/A	N/A
Tlacuilo-Parra, A	24/62	38.71%	10/62	16.13%	0.568 ± 0.13	0.632 ± 0.16	N/A	N/A	N/A	N/A
Mansouritorghabeh, H	5/14	35.71%	0/14	0%	1.09 ± 0.24	1.21 ± 0.15	0.99 ± 0.21	1.17 ± 0.30	N/A	N/A
Christoforidis, A	2/26	7.69%	0/13	0%	N/A	N/A	N/A	N/A	N/A	N/A
Rezaeifarid, M	N/A	N/A	N/A	N/A	0.908 ± 0.13	0.987 ± 0.19	0.889 ± 0.18	0.969 ± 0.11	N/A	N/A
Alioglu, B	N/A	N/A	N/A	N/A	0.52 ± 0.14	0.98 ± 0.23	N/A	N/A	N/A	N/A
Anagnostis, P	28/104	26.92%	10/50	20%	1.047 ± 0.17	1.065 ± 0.16	0.938 ± 0.16	1.015 ± 0.18	0.929 ± 0.16	1.018 ± 0.19
Wells, A. J	11/37	29.73%	6/37	16.22%	0.985 ± 0.20	1.082 ± 0.18	0.792 ± 0.22	0.905 ± 0.23	0.900 ± 0.20	1.051 ± 0.18
Eldash, H. H	5/30	16.67%	0/37	0%	0.63 ± 0.12	0.67 ± 0.11	N/A	N/A	N/A	N/A
Sossa Melo, C. L	28/89	31.46%	7/90	7.78%	N/A	1.06 (IQR:0.84–1.23)	N/A	N/A	N/A	N/A
Ashritha, A	8/38	21.05%	0/38	0%	0.487 (IQR:0.43–0.56)	0.549 (IQR:0.46–0.60)	N/A	N/A	N/A	N/A
Ekinci, O	10/41	24.39%	0/40	0%	0.92 (Min:0.83, Max:1.22)	1.01 (Min:0.01, Max:1.21)	0.92 (Min:0.71, Max:1.19)	1.12 (Min:0.94, Max:2.09)	0.94 (Min:0.70, Max:1.21)	1.12 (Min:0.94, Max:2.09)
El-Mikkawy, D. M. E	14/40	35%	0/10	0%	N/A	N/A	N/A	N/A	N/A	N/A
Ehsanbakhsh, A	18/57	31.58%	8/60	13.33%	− 1.1 ± 50.07	− 0.1 ± 78.20	N/A	N/A	− 0.0 ± 63.94	− 0.0 ± 33.98
Mohamed, H. R	11/39	28.21%	0/20	0%	0.61 ± 0.18	0.98 ± 0.10	N/A	N/A	N/A	N/A
Patel, G. R	16/39	15.38%	6/40	15%	0.56 (Min:0.19, Max:1.11)	0.67 (Min:0.21, Max:1.15)	N/A	N/A	0.49 (Min:0.27, Max:0.97)	0.64 (Min:0.31, Max:1.06)

*BMD* Bone mineral density, *H* Haemophilia, *C* Controls, *LS* Lumbar spine, *FN* Femoral neck, *TH* Total hip, *N/A* Non-available

### Quality assessment

The quality assessment of the included studies is presented in Additional file 1: Table S2, and 15 studies [18, 20–22, 24, 26–35] were clarified as high-quality while 4 [17, 19, 23, 25] studies were moderate-quality.

### Meta-analysis

**Fig. 2:**
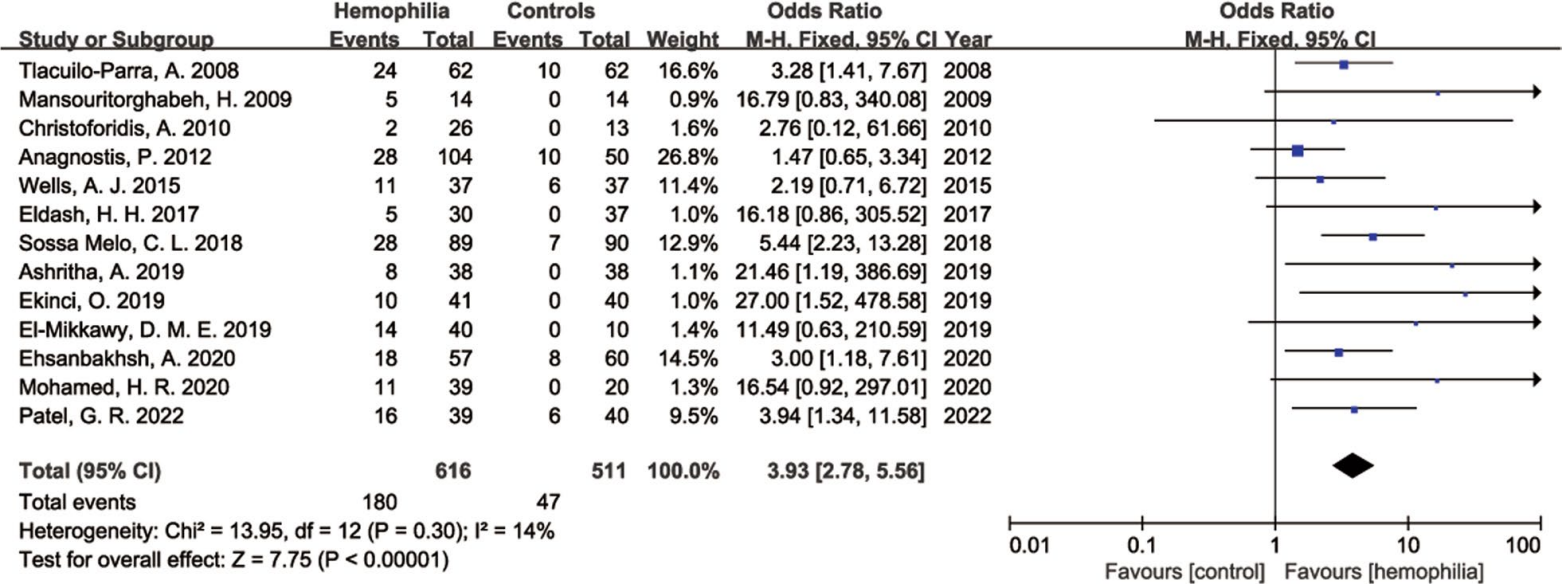
Forest plot of the risk of low BMD in PWH versus controls

As shown in Fig. 2, haemophilia was associated with an increased risk of low BMD (OR 3.93; 95% CI 2.78–5.56; *P* < 0.00001). Very low heterogeneity was observed among the evaluated studies (I^2^ = 14%, *P* = 0.30).

**Fig. 3:**
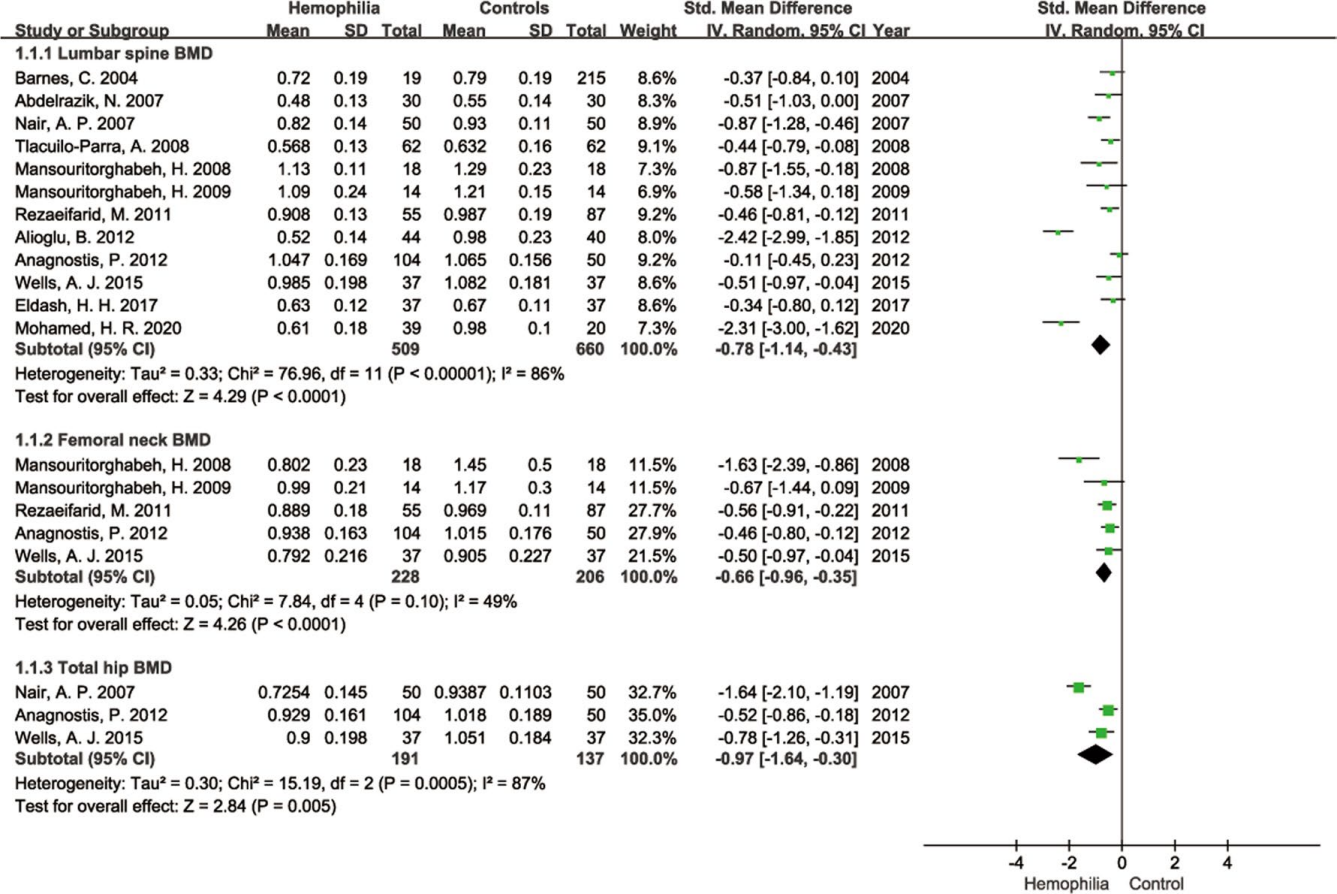
Forest plot of LS, FN, TH BMD in PWH versus controls

As illustrated in Fig. 3, overall results showed a significant reduction in LS BMD (SMD =  − 0.78; 95% CI − 1.14 to − 0.43; *P* < 0.0001; *I*^2^ = 86%), FN BMD (SMD =  − 0.66; 95% CI − 0.96 to − 0.35; *P* < 0.0001; *I*^2^ = 49%), TH BMD (SMD =  − 0.97; 95% CI − 1.64 to − 0.30; *P* = 0.005; *I*^2^ = 87%) in PWH when compared with controls. However, high heterogeneity was observed among the evaluated studies besides FN BMD.

### Subgroup analysis

We performed a subgroup analysis of the risk of low BMD and changes in LS BMD because these two analyses included enough studies for subgroup analysis. Subgroups classified by mean age (< 18 or > 18), region (Asia or others), country (developed country or developing country divided by the United Nations), and study quality (moderate or high) were analysed. All the results are shown in Tables 3 and 4. The subgroup analysis results indicated that the risk of low BMD was not statistically significant in developed countries (*P* = 0.10). Other meta-analysis results did not alter, and the findings were robust. However, we failed to reduce the high heterogeneity of LS BMD based on these factors.

**Table 3:** Subgroup analysis of the risk of low BMD in PWH versus controls

	Number of studies	Number of haemophilia	Number of controls	I^2^ (%)	Odds ratio (95% CI)	*P*
Overall	13	616	511	14	3.93 [2.78, 5.56]	*P* < 0.00001
Subgroup analysis						
Mean age						
> 18	5	253	201	36	2.75 [1.67, 4.54]	*P* < 0.0001
< 18	7	274	220	0	5.31 [2.98, 9.46]	*P* < 0.00001
Region						
Asia	5	189	192	11	5.42 [2.91, 10.09]	*P* < 0.00001
Others	8	427	319	19	3.38 [2.23, 5.14]	*P* < 0.00001
Country						
Developed country	3	167	100	0	1.73 [0.91, 3.30]	*P* = 0.10
Developing country	10	449	411	0	5.39 [3.54, 8.19]	*P* < 0.00001

**Table 4:** Subgroup analysis of LS BMD in PWH versus controls

	Number of studies	Number of haemophilia	Number of controls	*I*^2^ (%)	Random effects SMD (95% CI)	*P*
Overall	12	509	660	86	− 0.78 [− 1.14, − 0.43]	*P* < 0.0001
Subgroup analysis						
Mean age						
> 18	6	278	256	47	− 0.52 [− 0.77, − 0.26]	*P* < 0.0001
< 18	6	231	404	92	− 1.04 [− 1.74, − 0.33]	*P* = 0.004
Region						
Asia	5	181	209	88	− 1.04 [− 1.71, − 0.36]	*P* = 0.003
Others	7	328	451	81	− 0.60 [− 0.99, − 0.20]	*P* = 0.003
Country						
Developed country	3	160	302	2	− 0.28 [− 0.52, − 0.04]	*P* = 0.02
Developing country	9	349	358	87	− 0.95 [− 1.41, − 0.49]	*P* < 0.0001
Study quality						
High	9	396	355	76	− 0.62 [− 0.94, − 0.30]	*P* = 0.0001
Moderate	3	113	305	94	− 1.21 [− 2.30, − 0.11]	*P* = 0.03

*SMD* Standard mean difference, *CI* Confidence interval

### Sensitivity analysis

We performed a sensitivity analysis of the changes in LS and TH BMD because of their high heterogeneity. The leave-one-out approach was used for the sensitivity analysis of each outcome (Additional file 1: Table S3). The analysis results indicated that the meta-analysis results for LS BMD did not alter when each study was removed in turn and that the findings were robust. Unfortunately, we failed to reduce the high heterogeneity of LS BMD. And in the meta-analysis of TH BMD, the removal of study conducted by Nair et al. [19] (*I*^2^ = 0) suggested that this study could be the potential source of heterogeneity.

### Publication bias

Publication bias was investigated using the funnel plot. For the risk of low BMD, publication bias was suspected by observing the funnel plot (Additional file 1: Fig. S1). The result from Egger’s regression test suggested that there may be publication bias (*p* = 0.0091). Then, we performed the trim-and-fill correction procedure, and the meta-analysis results did not alter. The findings were robust (Additional file 1: Figs. S2 and S3).

## Discussion

We performed this systematic review and meta-analysis to evaluate the risk of low BMD and changes in BMD among PWH compared with the general population. The analysis incorporated data from 19 studies, involving 616 PWH for calculating the risk of low BMD, and 509 PWH for evaluating changes in BMD compared to control groups. Our observations revealed a significant increase in the risk of low BMD, as well as reduced BMD at various sites (LS, FN, and TH), among PWH when compared to the general population. In addition, subgroup analysis indicated a higher risk of low BMD among underage and Asian patients, compared to adults and individuals from other regions. Interestingly, our analysis did not find a statistically significant increase in the risk of low BMD in developed countries. And subgroup analysis of changes in BMD indicated that BMD reduced significantly among PWH regardless of age, region, or economic status.

To our knowledge, this is the first meta-analysis to comprehensively calculate the risk of low BMD in PWH. Additionally, this is the third meta-analysis that reports BMD changes in PWH compared with the general population. The first one [11], published in 2010, included seven studies comparing LS BMD between PWH and an age-matched general population. They found that LS BMD was significantly lower in both paediatric and adult PWH compared with controls. The second one [12], published in 2014, included ten and five studies to evaluate LS and FN BMD, respectively. They reported that PWH exhibited a severe reduction in both LS and FN bone mass, which may begin in childhood. But these two studies did not assess whether the extent of BMD decline fulfilled the criteria for low BMD. This meta-analysis addressed this gap by calculating the risk of low BMD among PWH.

Our findings revealed that the risk of low BMD in PWH was approximately four times higher compared to controls. The subgroup analysis results indicated that the risk of low BMD was not statistically significant in developed countries but BMD was reduced significantly. A multitude of factors may potentially contribute to this outcome. Developed countries boast superior social welfare and healthcare infrastructure [8]. Owing to preventive infusion of clotting factors, PWH can confidently engage in physical activities, consequently upholding normal BMD levels or decline to a degree that does not meet the criteria for low BMD. Moreover, only 3 studies [23, 26, 27] were analysed to evaluate the risk of low BMD in developed countries, which may lead to bias.

For the changes in BMD, we included 12, 5, and 3 studies to evaluate the BMD of different sites (LS, FN, and TH) in PWH compared with the controls, respectively. We reached the same conclusion as Iorio et al. [11] and Paschou et al. [12]. Moreover, not only LS and FN but also TH BMD was reduced significantly. Regrettably, an extreme degree of heterogeneity was also observed among the evaluated studies of BMD. Thus, we carried out a subgroup analysis and sensitivity analysis. For LS BMD, the subgroups were classified by mean age, region, and country. The results showed that Asian and underage PWH had lower LS BMD than individuals in other regions and adults. But we failed to reduce the high heterogeneity of LS BMD by subgroup analysis and sensitivity analysis. And in the meta-analysis of TH BMD, the removal of study conducted by Nair et al. [19] (*I*^2^ = 0) indicated that this study could be the potential source of heterogeneity. This study focused on PWH in India. And the other two studies were from Greece [26] and the United Kingdom [27]. This study recruited PWH with a significantly lower BMI compared to the other two studies. Among non-obese individuals, elevated BMI correlated with gradual enhancements in BMD [36]. Besides, a majority of patients in this Indian study exhibited chronic arthritis, which hampers regular exercise. Physical activity is known to promote BMD enhancement [37]. Calcium intake among Indians falls significantly below daily requisites due to dietary habits [38]. We can even find that the BMD of the Indian control group is much lower than that of the Greek and British control groups. These factors could potentially hinder BMD from reaching its peak, thereby contributing to heterogeneity.

The underlying mechanism of how haemophilia affects BMD is not well understood. Several reasons may explain the high prevalence of low BMD in PWH. Firstly, FVIII affected bone resorption. The receptor activator of nuclear factor kappa-B ligand (RANKL) binds to RANK and promotes osteoclastogenesis. Osteoprotegerin (OPG) competitive binding with RANKL to inhibit osteoclastogenesis [39, 40]. FVIII-vWF complex inhibits osteoclastogenesis RANKL induced and enhances the inhibitory effect of OPG [41]. But FVIII and vWF do not inhibit RANKL alone. The activity of FVIII is low in PWH, so they do not have enough FVIII-vWF complex to inhibit osteoclastogenesis RANKL induced, leading to bone resorption. Secondly, FVIII affects bone formation. Weitzmann et al. [42] examined imaging examination, quantitative bone histomorphometry, and serum bone markers of FVIII-knockout mice. The results showed that trabecular bone accretion of male FVIII-knockout mice lagged significantly between 2 and 6 months of age and the bone formation markers (N-terminal propeptide of type 1 procollagen) were decreased. Besides, osteoblasts can express thrombin receptors [43] and thrombin can stimulate osteoblast proliferation [44]. Lack of FVIII or FIX can result in impaired FX activation and failure of thrombin production, resulting in reduced bone formation. Finally, exercise is reduced. Adolescence is a critical period for BMD growth. More than 94% bone mass is gained before the age of 16 [45]. Several randomized controlled trials [46, 47] have shown that physical activity is beneficial to the increase of BMD in adolescence. However, PWH engage in less physical activity due to pain and other reasons [48, 49]. Lack of exercise affects bone mass and results in BMD not reaching its peak. Multiple studies [37, 50] have suggested that sportive activity had a positive impact on BMD in PWH.

In recent years, advancements in treatment have enabled PWH to attain a nearly normal quality of life and life expectancy. Early prophylactic infusion of coagulation factors is recommended [1]. Prophylactic treatment can alleviate pain, decrease the frequency of bleeding, and safeguard joint function in both adults and children [51, 52]. Therefore, individuals are more inclined to participate in social activities and physical exercises to preserve bone health and enhance their quality of life. Meanwhile, age-related diseases should be paid attention to in clinical treatment, such as low BMD. Osteoporotic fractures are among the complications of osteoporosis, and the prognosis is particularly grim for men [53]. The incidence of osteoporotic fractures was significantly higher in PWH. A 10-year retrospective analysis conducted within a single institution revealed that PWH exhibited a greater prevalence of fractures compared to the general population [54]. Additionally, arthropathy is a prevalent and severe complication due to recurrent joint bleeding in PWH, leading to chronic pain and reduced quality of life. Joint replacement is seen as the best option for patients with advanced haemophilia [55]. However, individuals with osteoporosis are at a higher risk of experiencing adverse outcomes, including intraoperative and postoperative periprosthetic fractures [56].

For PWH, our concerns should extend beyond bleeding and osteoarthritis to encompass BMD starting at a young age. The majority of research was still in the stage of animal experiments. If the mechanism of osteoporosis in haemophiliacs is verified in humans, physicians can employ targeted medications for bone resorption or bone formation to enhance bone density and prevent fractures. Moreover, there is limited research on medical therapy for low BMD in PWH. Only one study [57] had evaluated the efficacy of ibandronate for osteoporosis in PWH so far. More high-quality cohort studies should be carried out to guide clinical medication in the future.

We acknowledged some limitations of our meta-analysis. Firstly, the published studies are insufficient for subgroup analysis to find the source of heterogeneity. Secondly, this meta-analysis had publication bias, though we performed the trim-and-fill correction procedure, and the outcome did not alter. Thirdly, because haemophilia is a rare disease, the number of cases included in this study was relatively small. Fourthly, the wide time frame of the included studies including patients with different treatment possibilities may impact results of the analysis. Finally, few studies reported the severity of haemophilia, so we failed to group haemophilia patients by severity.

## Conclusion

The results of this meta-analysis indicated an elevated risk of low BMD in PWH. In addition, the prevalence of low BMD appeared to be higher among underage and Asian patients compared to adults and other regions. However, the risk of low BMD did not increase significantly in developed countries. And BMD was reduced in PWH, regardless of region or age. In clinical treatment, for PWH, we should not only be concerned about bleeding and osteoarthritis but also BMD from an early age. It is beneficial to reduce the risk of osteoporotic fractures and periprosthetic fractures during joint replacement for end-stage haemophilic osteoarthritis. In addition, we should clarify the mechanism of bone density reduction and determine targeted treatment methods for osteoporosis in PWH.

### Supplementary Information

**Additional file 1**. **Table S1** Search strategy. **Table S2** Quality assessment. **Table S3** Sensitivity analysis. **Fig. S1** Funnel plots. **Fig. S2** Egger’s regression test. **Fig. S3** Trim-and-fill correction.

## Data Availability

The data that supports the findings of this study are available in supplementary material of this article.

## Abbreviations

PWH: Patients with haemophilia
BMD: Bone mineral density
DXA: Dual X-ray absorptiometry
BMI: Body mass index
OR: Odds ratios
CI: Confidence intervals
SMD: Standardized mean differences
SD: Standard deviation
LS: Lumbar spine
FN: Femoral neck
TH: Total hip
RANKL: The receptor activator of nuclear factor kappa-B
OPG: Osteoprotegerin
