# Supplementary material for: Risk of low bone mineral density in patients with haemophilia: a systematic review and meta-analysis
Source: J Orthop Surg Res. 2024 Jan 11;19:52. doi: 10.1186/s13018-023-04499-6 (PMC10782745; doi:10.1186/s13018-023-04499-6)

Supplementary Material

**Table S1-Search strategy used**

| **PubMed** | ((("Hemophilia A"[Mesh]) OR ("Hemophilia B"[Mesh])) OR ((hemophilia[Title/Abstract]) OR (haemophilia[Title/Abstract]))) AND ((("Bone Density"[Mesh]) OR ("Osteoporosis"[Mesh])) OR ((bone mineral density[Title/Abstract]) OR (Osteopenia[Title/Abstract]))) |
| --- | --- |
| **Embase** | #1 'hemophilia'/exp 48768  #2 haemophilia:ab,ti OR 'hemophilia a':ab,ti OR 'hemophilia b':ab,ti 29763  #3 #1 OR #2 50434  #4 'bone density'/exp OR 'osteoporosis'/exp OR 'osteopenia'/exp 220435  #5 'bone mineral density':ab,ti 65909  #6 #4 OR #5 224620  #7 #3 AND #6 444 |
| **Cochrane Library** | #1 MeSH descriptor: [Hemophilia A] explode all trees 472  #2 MeSH descriptor: [Hemophilia B] explode all trees 121  #3 (hemophilia):ti,ab,kw 1657  #4 (haemophilia):ti,ab,kw 1657  #5 #1 OR #2 OR #3 or #4 1657  #6 MeSH descriptor: [Bone Density] explode all trees 4897  #7 MeSH descriptor: [Osteoporosis] explode all trees 4383  #8 (bone mineral density):ti,ab,kw 9211  #9 (Osteopenia):ti,ab,kw 1342  #10 #6 OR #7 OR #8 OR #9 12518  #11 #5 AND #10 4 |
| **Web of Science** | #1 (((TS=(Hemophilia A)) OR TS=(Hemophilia B)) OR TS=(hemophilia)) OR TS=(haemophilia) 32154  #2 (((TS=(Bone Density)) OR TS=(Osteoporosis)) OR TS=(bone mineral density)) OR TS=(Osteopenia) 181714  #3 #1 AND #2 225 |

**Table S2-Quality assessment of studies included in this meta-analysis by Newcastle-Ottawa Scale**

| **Study** | **Selection** | | | | **Comparability** | **Exposure** | | | **Total score** | **Quality grade** |
| --- | --- | --- | --- | --- | --- | --- | --- | --- | --- | --- |
|  | **Is the case definition adequate** | **Representativeness of the cases** | **Selection of Controls** | **Definition of Controls** | **Comparability** | **Ascertainment of exposure** | **Same method of ascertainment for cases and controls** | **Non-Response rate** |  |  |
| Barnes, C. 2004 | ★ |  |  | ★ | ★★ | ★ | ★ |  | 6 | moderate |
| Abdelrazik, N. 2007 | ★ | ★ | ★ | ★ | ★★ | ★ | ★ | ★ | 9 | high |
| Nair, A. P. 2007 | ★ |  | ★ | ★ | ★ | ★ | ★ |  | 6 | moderate |
| Mansouritorghabeh, H. 2008 | ★ |  | ★ | ★ | ★★ | ★ | ★ |  | 7 | High |
| Tlacuilo-Parra, A. 2008 | ★ | ★ | ★ | ★ | ★★ | ★ | ★ | ★ | 9 | High |
| Mansouritorghabeh, H. 2009 | ★ | ★ | ★ | ★ | ★★ | ★ | ★ | ★ | 9 | High |
| Christoforidis, A. 2010 | ★ | ★ |  | ★ | ★ | ★ | ★ |  | 6 | moderate |
| Rezaeifarid, M. 2011 | ★ | ★ | ★ |  | ★ | ★ | ★ | ★ | 7 | High |
| Alioglu, B. 2012 | ★ |  |  | ★ | ★ | ★ | ★ |  | 5 | moderate |
| Anagnostis, P. 2012 | ★ | ★ |  | ★ | ★★ | ★ | ★ |  | 7 | High |
| Wells, A. J. 2015 | ★ |  | ★ | ★ | ★★ | ★ | ★ |  | 7 | High |
| Eldash, H. H. 2017 | ★ | ★ |  | ★ | ★ | ★ | ★ | ★ | 7 | High |
| Sossa Melo, C. L. 2018 | ★ |  | ★ | ★ | ★★ | ★ | ★ |  | 7 | High |
| Ashritha, A. 2019 | ★ | ★ |  | ★ | ★★ | ★ | ★ | ★ | 8 | High |
| Ekinci, O. 2019 | ★ | ★ | ★ | ★ | ★★ | ★ | ★ | ★ | 9 | High |
| El-Mikkawy, D. M. E. 2019 | ★ | ★ | ★ | ★ | ★ | ★ | ★ | ★ | 8 | High |
| Ehsanbakhsh, A. 2020 | ★ | ★ | ★ | ★ | ★★ | ★ | ★ | ★ | 9 | High |
| Mohamed, H. R. 2020 | ★ |  | ★ | ★ | ★★ | ★ | ★ | ★ | 8 | High |
| Patel, G. R. 2022 | ★ | ★ |  | ★ | ★★ | ★ | ★ | ★ | 8 | high |

**Table S3-Results of sensitivity analysis**

1. **Sensitivity analysis for LS BMD**

| **Study** | **SMD (95%CI)** | ***P*** | **I^2^** |
| --- | --- | --- | --- |
| Barnes, C. 2004 | -0.82 [-1.21, -0.44] | *P*<0.0001 | 87 |
| Abdelrazik, N. 2007 | -0.81 [-1.20, -0.42] | *P*<0.0001 | 87 |
| Nair, A. P. 2007 | -0.78 [-1.17, -0.38] | *P=*0.0001 | 87 |
| Tlacuilo-Parra, A. 2008 | -0.82 [-1.22, -0.42] | *P*<0.0001 | 87 |
| Mansouritorghabeh, H. 2008 | -0.78 [-1.16, -0.40] | *P*<0.0001 | 87 |
| Mansouritorghabeh, H. 2009 | -0.80 [-1.18, -0.42] | *P*<0.0001 | 87 |
| Rezaeifarid, M. 2011 | -0.82 [-1.22, -0.42] | *P*<0.0001 | 87 |
| Alioglu, B. 2012 | -0.62 [-0.88, -0.35] | *P*<0.00001 | 73 |
| Anagnostis, P. 2012 | -0.85 [-1.23, -0.47] | *P*<0.00001 | 85 |
| Wells, A. J. 2015 | -0.81 [-1.20, -0.42] | *P*<0.0001 | 87 |
| Eldash, H. H. 2017 | -0.83 [-1.21, -0.44] | *P*<0.0001 | 87 |
| Mohamed, H. R. 2020 | -0.66 [-0.97, -0.34] | *P*<0.0001 | 81 |

**2.** **Sensitivity analysis for TH BMD**

| **Study** | **SMD (95%CI)** | ***P*** | **I**^2^ |
| --- | --- | --- | --- |
| Nair, A. P. 2007 | -0.61 [-0.89, -0.33] | *P*<0.0001 | 0 |
| Anagnostis, P. 2012 | -1.21 [-2.06, -0.37] | *P*=0.005 | 85 |
| Wells, A. J. 2015 | -1.07 [-2.17, 0.03] | *P*=0.06 | 93 |

**Fig.S1-Funnel plots of risk of low BMD in PWH vs controls**


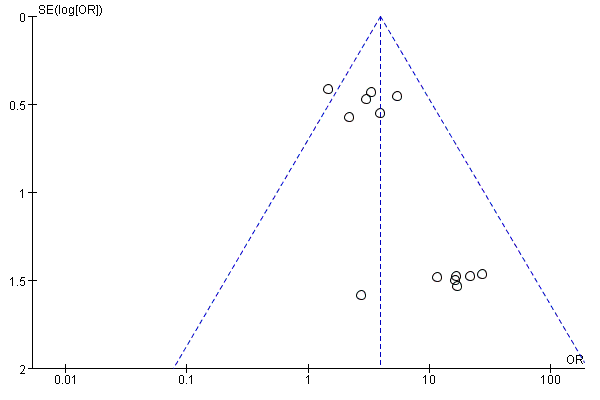


**Fig.S2-result of Egger’s regression test**


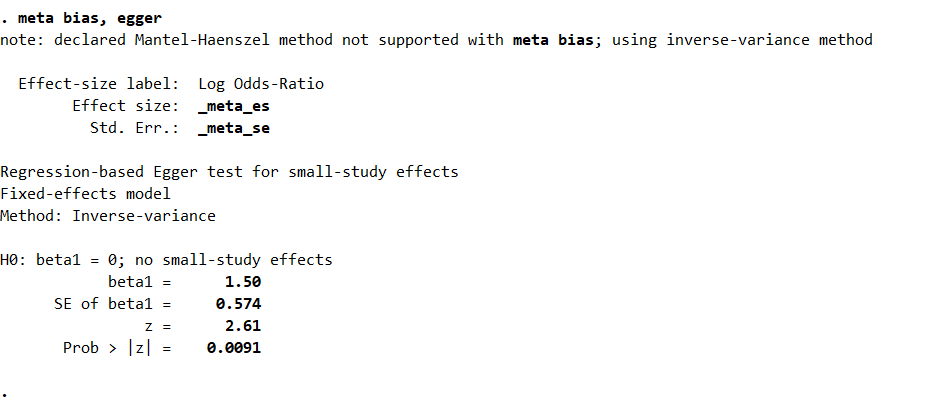


**Fig.S3-result of the trim-and-fill correction**


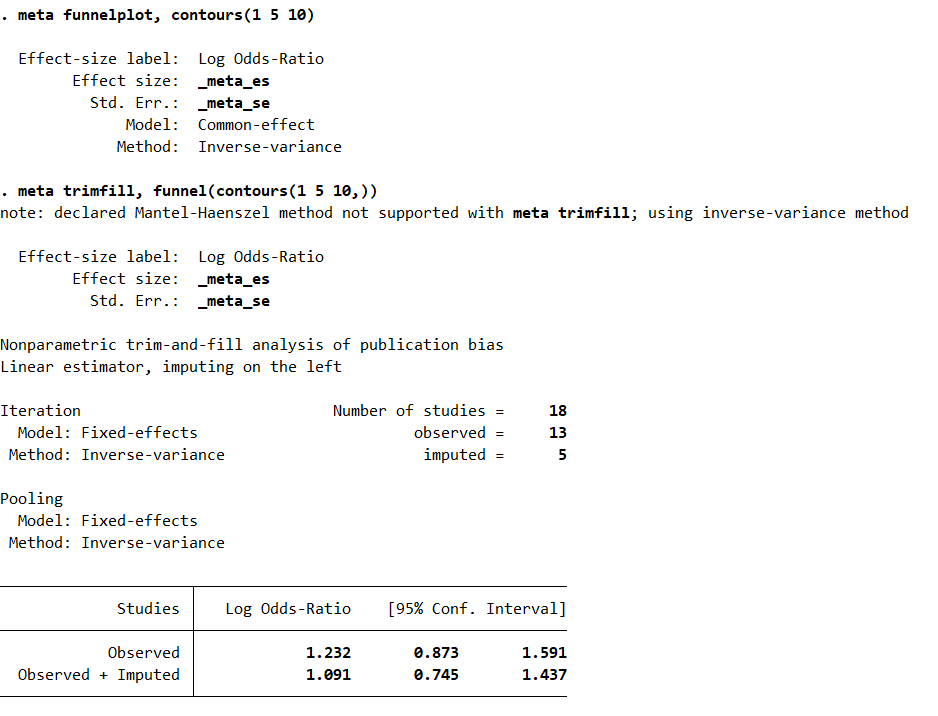

Supplement: Supplementary file 1 — Additional file 1. Table S1 Search strategy. Table S2 Quality assessment. Table S3 Sensitivity analysis. Fig. S1 Funnel plots. Fig. S2 Egger’s regression test. Fig. S3 Trim-and-fill correction. [file 13018_2023_4499_MOESM1_ESM.docx]
